# Education, training and technological innovation, key components of the ESTES-NIGHTINGALE project cooperation for Mass Casualty Incident preparedness in Europe

**DOI:** 10.1007/s00068-022-02198-1

**Published:** 2022-12-13

**Authors:** Carlos Yánez Benítez, Jonathan Tilsed, Eric S. Weinstein, Marta Caviglia, Simon Herman, Carl Montán, Gerhard Achatz, Joe Cuthbertson, Luca Ragazzoni, Evangelos Sdongos, Itamar Ashkenazi, Roberto Faccincani

**Affiliations:** 1 European Society of Trauma and Emergency Surgery (ESTES), St. Pölten, Austria; 2 Center for Research and Training in Disaster Medicine, Humanitarian Aid, and Global Health (CRIMEDIM), Università del Piemonte Orientale, Novara, Italy; 3 ASTRIAL GmbH, Berlin, Germany; 4 General and Acute Care Surgery, San Jorge University Hospital, Huesca, Spain; 5 Surgery Health Care Group, Hull University Teaching Hospitals NHS Trust, Hull, UK; 6 Department for Sustainable Development and Ecological Transition, Università del Piemonte Orientale, Vercelli, Italy; 7 Department of Traumatology, University Medical Centre, Ljubljana, Slovenia; 8 Vascular and General Surgeon, Karolinska University Hospital, Stockholm, Sweden; 9 Department for Orthopedics and Trauma Surgery, Reconstructive and Septic Surgery, Sportstraumatology, German Armed Forces Hospital Ulm, Oberer Eselsberg 40, 89081 Ulm, Germany; 10 Monash University Disaster Resilience Initiative, Monash University, Melbourne, Australia; 11 Department of General Surgery, Rambam Health Care Campus, Haifa, Israel; 12 Emergency Department, Humanitas Mater Domini, Castellanza, Italy

**Keywords:** Terrorism, Triage, Mass casualty, Disaster medicine, Technological innovation

## Abstract

Disasters induced by extreme weather events and terrorism-related activities, causing mass casualty incidents (MCIs) in Europe, are expected to increase in the upcoming years. This challenging scenario demands a high level of readiness and coordinated multi-disciplinary response to reduce morbidity and mortality. The European Society of Trauma and Emergency Surgery (ESTES) is one of the 23 partners of the European-funded project Novel Integrated Toolkit for Enhanced Pre-Hospital Life Support and Triage in Challenging and Large Emergencies (NIGHTINGALE), whose primary objective is to promote the exchange in experiences and define the best practices among first responders. Additionally, the project promotes multi-disciplinary and multi-institutional efforts to achieve technological innovation that will enhance preparedness in MCI management. This manuscript aims to describe the challenges of MCI triage, the education and training programs for MCI response in Europe, and the technological innovation that may aid optimal response. These three elements were discussed by ESTES Disaster and Military Surgery Section members during the German Society for Trauma Surgery session at the ECTES 2022 in Oslo “TDSC^®^ and beyond: ideas and concepts for education and training in Terror Preparedness”, additionally the manuscript describes the first steps of the cooperation between ESTES and the rest of the NIGHTINGALE consortium.

## Background

**Fig. 1:**
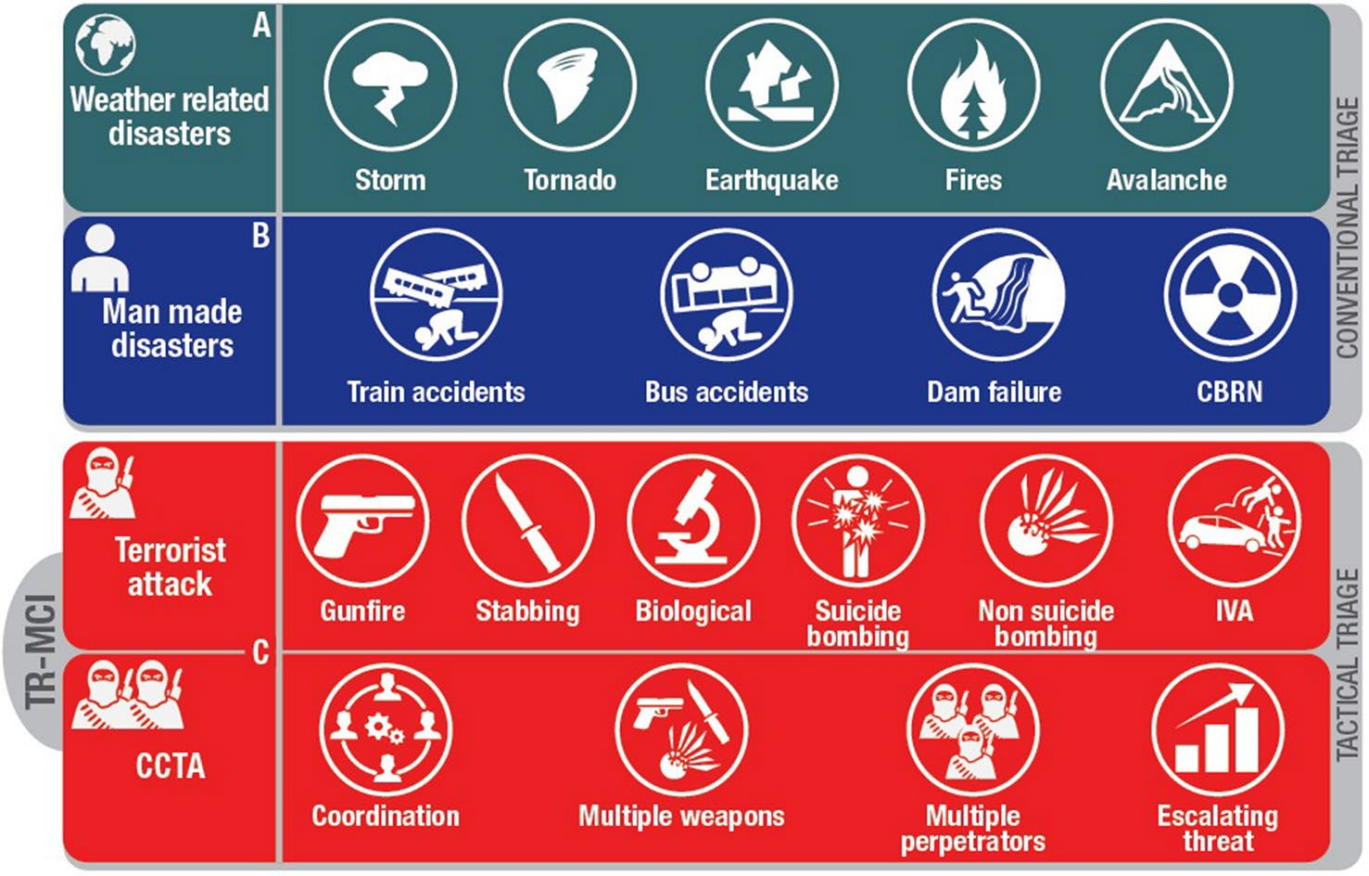
The ESTES-NIGHTINGALE project cooperation focuses on several types of MCIs. (TR-MCI Terrorist-related MCI). **A** Weather-related disaster. **B** Nonintentional man-made Mass Casualty Incident. **C** Complex and Coordinated Terrorist Attacks (CCTAs): involving orchestrated and coordinated tactics, incidents produced by various types of weapons, or perpetrated by multiple terrorists in several sites simultaneously or in an escalating pattern

Mass Casualty Incidents (MCIs) are situations where the supply of immediately available resources is insufficient to meet the demand for medical care of the injured. It is not related to any specific number of injured individuals or any level of resources but refers to the balance between resources and needs [1]. Although MCIs can be the product of natural disasters, such as earthquakes or extreme weather events, recent reports suggest that climate change and global warming may increase the characteristics and frequency of weather-related disasters [2]. Non-weather-related disasters can be either non-intentional, such as bridge/building collapse or train/plane crash, or intentional, termed terrorist-related MCI (Fig. 1A–C). Terrorist attacks peaked worldwide in 2015 and have gradually decreased in the last few years. Despite this trend, it is still a subject of concern, with 57 terrorist attacks reported in Europe in 2020 [3, 4].

European cities have suffered several attacks in the last decades, causing numerous deaths and wounded civilians [5–9]. These incidents have involved single or multiple perpetrators using numerous weapons (bombs, mass shootings, vehicle-ramming attacks, etc.). Some of these incidents have been classified as Complex and Coordinated Terrorist Attacks (CCTAs), since these involve coordinated tactics and simultaneous use of various weapons, most carried out by multiple perpetrators on several sites simultaneously or in an escalating pattern [10] (Fig. 1C). Tin et al. in 2021, based on their examination of the Global Terrorism Database, determined that the most common weapons used in terrorist-related MCI in the last 50 years have been explosives (48.7%), followed by firearms (26.77%), a combination of weapons (7.23%), and incendiary weapons (6.39%) [11]. However, recent terrorist attacks have evolved in form and complexity. There is a growing trend to use unsophisticated methods of attack, such as mass stabbings and intentional vehicle assaults. In addition to this change in the forms of attacks, there is also a change in frequency. The public health measures introduced to limit the spread of the Severe Acute Respiratory Syndrome Coronavirus 2 (SARS-CoV-2 virus), including restrictions on air travel, mass gatherings, and other "lockdown measures" in major European cities, may have reduced terrorism-related incidents [3, 12]. The coronavirus disease 19 (COVID-19) pandemic disrupted terrorist organizations' capacity to conduct attacks. Interpol reports that the impact of COVID-19 on the global economy could have indirectly affected the funding of terrorist organizations [13]. However, experts suggest that this reduction in terrorist activity is a temporary phenomenon, and a post-pandemic threat increase is expected [14, 15].

With this complex scenario, the European Union (EU) faces the challenge of enhancing preparedness among law enforcement agencies, first responders, and medical care professionals. NIGHTINGALE is a large-scale research and innovation project funded by the EU Horizon 2020 program [16]. This program is the EU's flagship research and innovation plan that delivers financial support to secure Europe's competitiveness in three aspects: science, industrial leadership, and social challenges. NIGHTINGALE aims to enhance European first responder’s preparedness and efficiency through multi-institutional cooperation and technological innovation.

**Fig. 2:**
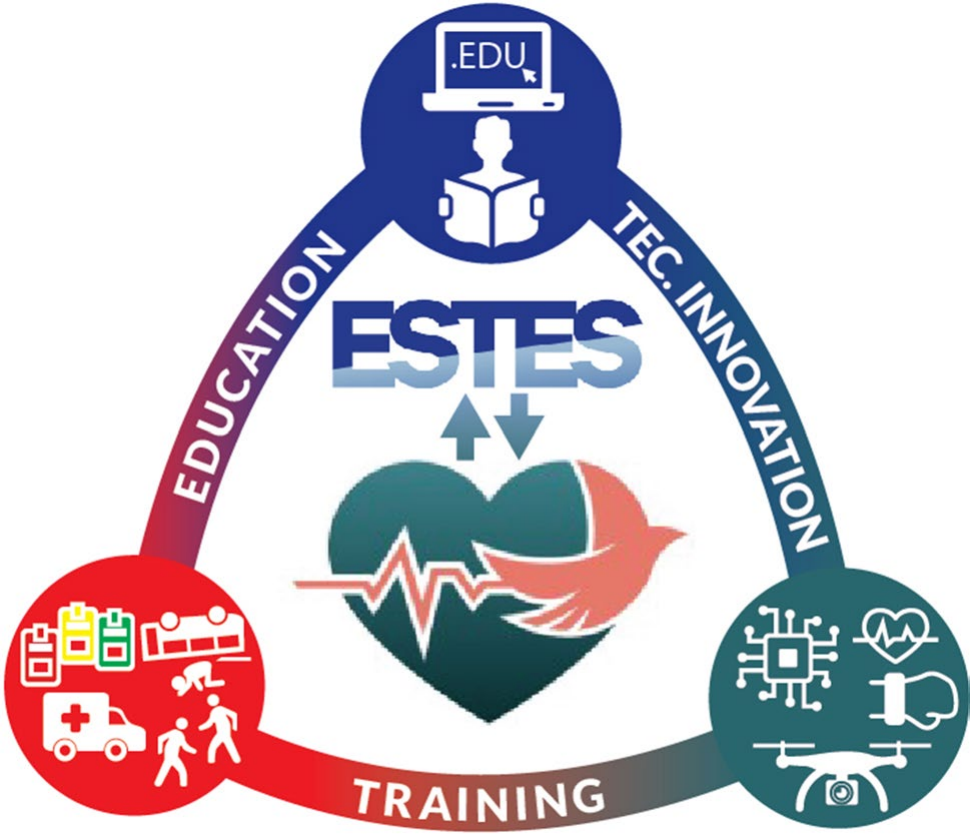
Integration of education, training, and technological innovations as key elements of the ESTES-NIGHTINGALE project cooperation

The European Society of Trauma and Emergency Surgery (ESTES) is a multinational academic society that deals with trauma and emergency surgery providing a platform for the research, education, training, and dissemination of information in Europe and other regions through associated societies. As one of the 23 NIGHTINGALE project partners, ESTES collaboration seeks to find the optimal means to enhance preparedness and optimize the response to all types of MCIs. This manuscript aims to briefly describe the management problems identified during MCI triage, training and education deficiencies, and technological innovation shortcomings of the tools developed by the consortium to achieve this objective (Fig. 2).

## Methods

In preparation for the German Society for Trauma Surgery session during the ECTES 2022 in Oslo, "TDSC^®^ and beyond: ideas and concepts for education and training in Terror Preparedness", in April 2022, a selected workgroup of international members of the Disaster and Military Surgery Section of ESTES with experience managing MCIs performed a literature review on PubMed, EMBASE databases and grey literature from 2000 to 2022. The study aimed to identify updated publications and knowledge gaps related to prehospital triage, training, and education, and innovative technological tools for MCI management focused on terrorism-related MCIs. Subsequently, the workgroup performed online discussions of the identified literature followed by live discussions during the congress with the participation of the session panelists and assistants, contrasting the literature findings with the panel's personal experience, converging on the strategies and tools to help attain optimal preparedness and response during MCIs. The workgroup discussions focused on MCI's prehospital triage, education and training programs in Europe, and innovative technological resources for first responders and health providers during MCI management.

## Challenges of MCI triage

Triage is a critical component of the MCI medical management, allowing for sorting and categorizing the injured on-site according to their immediate needs. This process evaluates the need for on-site essential Life-Saving Interventions (LSIs) and transport priority to the most appropriate medical facility. The main problem identified by the workgroup is a need for clinical validation of triage systems for the different MCI scenarios [17]. Most triage systems apply a combination of physiological variables and algorithms to classify each patient into a category to determine LSIs and priority transport. Commonly used are SIEVE triage, triage SORT the Simple Triage and Rapid Treatment (START), JumpSTART algorithm for pediatric victims, and the Sort, Assess, Life-saving interventions, and Treatment / Transport (SALT), among others [18, 19].

Each Emergency Medical Service or First Response Agency has its preferred triage method. Khorram–Manesh et al. highlight the need for a standard or universal triage system specific to MCI management to enable continuity in practice and interoperability among participating agencies [20]. Experience from terrorist-related MCIs reveals that most responders rely more on their clinical judgment than on the strict detection of physiologic parameters [21–23]. During the ESTES’s Disaster and Military Surgery Section session group discussion, several members provided feedback of their personal experience with warm zone triage during CCTAs, considering it should be rapid, straightforward, and reproducible, depending on more visual parameters, such as neurologic or physical activity, airway patency, identification of breathing patterns, such as gasping or rapid shallow breathing, rough estimation of blood loss and active hemorrhage, as well as the anatomic location of injuries. A simplified dichotomous scale of absolute emergency/relative emergency seems adequate for CCTAs [24–26]. Furthermore, during active shooter incidents, warm zone triage should be performed by adequately trained Tactical Paramedics and Rescue Task Forces that have completed a focused training program [27].

The ESTES-NIGHTINGALE cooperation seeks to achieve and validate a reliable scientific consensus for identifying the optimal triage method for all MCI scenarios. The joint workgroup suggests using “universal denominators” among the most common prehospital triage systems to set the basis for optimizing the prehospital triage process. This objective will be achieved by performing discussion and consensus among specifically created international workgroups of experts with clinical experience managing MCIs. ESTES’s Disaster and Military section members are well suited for this task considering the extensive clinical experience of the section members, who aim to promote knowledge and quality of care in emergency and trauma surgery.

## Education and training

Education and training for MCI management should be continuous and dynamic, a process incorporating simulation and tabletop exercises involving all levels of care, from first responders to definitive care provided by the trauma teams. It should also include coordination with the non-medical agencies involved, such as Firefighters, Police, and Civil Protection. The workgroup identified through a literature review and clinical practice of personal experience the need to develop a strategy to implement both a mindset and specific skillset to respond to MCIs, which are different from those required for daily clinical practice. The workgroup acknowledged that most of Europe's healthcare professionals lack the necessary exposure to maintain proficiency in treating penetrating trauma produced by firearms and blast injuries. Therefore, education programs centered on clinical decision-making during MCIs and competence to treat such patients are crucial elements of ESTES' contribution to NIGHTINGALE. Bieler et al. describe a novel concept of Tactical Abbreviated Surgical Care (TASC) that prioritizes an abbreviated surgery with control of bleeding and contamination, not because of physiologic parameters of the victim but rather considering the optimal use of surgical resources when hospitals providing care are overwhelmed by the number of victims during MCIs [28]. This unconventional approach based on the situation and not on the individual patient appears adequate for CCTA scenarios and should be incorporated in the training of the clinicians involved.

ESTES' contribution to the NIGHTINGALEs consortium's educational and training needs is based on the promotion of content directed to physicians, prehospital first responders, and trauma teams treating casualties of MCIs. This will be achieved by supporting specific courses that teach skills and decision-making during MCIs. The Medical Response to Major Incidents and Disasters Course (MRMI^®^), the Terror and Disaster Surgical Care Course (TDSC^®^), and the American College of Surgeons Stop the Bleed campaign are three educational and training activities endorsed by ESTES related to the care of casualties during MCIs. MRMI^®^ course is an essential NIGHTINGALE project partner, developed in 2009 by a group of international experts from ESTES's Disaster & Military Surgery Section, which has trained over 7000 participants from 28 countries since 2009 [29]. MRMI^®^ employs simulation exercises to teach optimal response during MCIs to the complete response chain. In addition, the program uses a simulation model that allows the education and training of prehospital and hospital staff, focusing on the assessment, planning, and preparation for major incident response among all the responding agencies [29–32]. TDSC^®^, launched in 2016 in cooperation with the Deployment, Disaster, and Tactical Surgery Working Group of the German Trauma Society, incorporates the difference between conventional mass casualty events and CCTAs [33]. This unique program uses a tabletop simulation exercise to teach quick decision-making with limited information [34]. Franke et al. also highlight the possibility of using both ATLS^®^ and the TDSC^®^ principles for the optimal initial intrahospital care of victims of terrorism-related mass casualty events [35].

The Stop the Bleed campaign launched by the American College of Surgeons in 2015 seeks to enhance survivability from mass casualty shootings by teaching citizens and immediate first responders’ basic concepts to treat life-threatening hemorrhage with three measures: local compression, hemostatic dressings and tourniquet application [36, 37]. It outlines actions for first responders after active shooter events, summarized by the THREAT acronym (T-threat suppression, H-hemorrhage control, RE-rapid extrication, and T-transport to definitive care) [38, 39]. The program was officially endorsed by ESTES and launched during the 2022 clinical congress in Oslo. The aim is to increase the number of instructors capable of teaching these simple techniques to the non-medical community and first responders potentially involved in MCIs. ESTES Disaster and Military care members are qualified clinicians committed to trauma care and dedicated and experienced in education among professionals treating MCI victims. This synchronism between clinical experience and the educational background of ESTES members makes this educational contribution to NIGHTINGALE optimal.

## Technological innovation

Technological innovation for MCI management is a rapidly evolving field influenced by the miniaturization and robustness of electronic devices, the development of specialized sensors, advances in telecommunications, and artificial intelligence software. However, the workgroup identified limitations in implementing these innovations, interfering with a coordinated, reliable, and cost-efficient response. The workgroup underlined the importance of data's reliability, validity, and accuracy, which should be safely stored, avoiding overlapping personal data or misuse. ESTES members provide crucial feedback based on their personal experience to the NIGHTINGALE project technological innovation team to help the consortium partners overcome the identified limitations related to the clinical implementation of the tools. Additionally, the ESTES workgroup shares its clinical expertise with the technology development partners to facilitate the elaboration of specialized tools for electronic patient identification and electronic web-based triage tags that will improve communication and information transfer from the pre-hospital to the receiving hospitals. They will also provide feedback for the creation of reliable, low-power consumption, and ergonomic wearable health devices with sophisticated sensors that will enable continuous ambulatory monitoring of vital signs. These will allow real-time monitoring of heart rate, blood pressure, respiratory rate, blood oxygen saturation, and body temperature, facilitating field triage and decision-making from the clinical and the logistical point of view. Additionally, ESTES members work alongside other members of the consortium to attempt to develop a governance framework for the obtained data.

ESTES members also contribute to tools under development, such as crewless aerial vehicles, frequently referred to as "drones," equipped with high-resolution cameras. These will be deployed ahead of the ground response when the location is remote or unsafe due to ongoing threats with firearms and explosives or in cases of CBRN hazard events. Additionally, the sensor-equipped drones will transfer real-time images to the MCI Command and Control Center, allowing prehospital response teams to organize within the scene efficiently. Finally, another element where ESTES provides constructive input is in developing software and artificial intelligence tools that will assist the first responders and the command centers in rapid decision-making both from the clinical and the logistical point of view. A detailed description of the extensive technological development of the NIGHTINGALE project technical teams is beyond the scope of this manuscript. Still, the ESTES-NIGHTINGALE workgroup group proposes that the specialized tools will be:

- Inexpensive
- Easy for agencies to deploy, maintain and store
- Easy for agencies to educate, train and maintain competencies
- Ubiquitous, that is to be available to be deployed anywhere in a jurisdiction or an area (one device in an area, kilometers away serves little purpose)
- Able to produce meaningful data that is superior to data already available

ESTES contribution is invaluable to the technology partners that lack clinical knowledge and personal experience treating casualties during MCIs.

## Conclusions

Natural and extreme weather disasters will persist and most likely increase with global warming. Likewise, terrorist-related MCIs in Europe are expected to increase in the post-pandemic era. The ESTES-NIGHTINGALE project cooperation offers a groundbreaking opportunity to modernize and enhance European first response to these challenges. ESTES Disaster and Military Surgery Section members work with other NIGHTINGALE project associates sharing clinical expertise and personal experiences to optimize Europe's response to MCIs. These efforts include educational and training programs and technological innovation through multi-disciplinary and multi-institutional enterprises, intending to reduce morbidity and mortality by enhanced preparedness to respond to these demanding MCI scenarios.

## Data Availability

The primary data that supports the study findings is not publicly available. However, the authors can share data upon reasonable request and permission of the ESTES-NIGHTINGALE scientific coordinators.

## Abbreviations

MCI: Mass Casualty Incidents
ESTES: European Society of Trauma and Emergency Surgery
EU: European Union
NIGHTINGALE: Novel Integrated Toolkit for Enhanced Pre-Hospital Life Support and Triage in Challenging and Large Emergencies
CCTA: Complex and coordinated terrorist attack
CBRN: Chemical, biological, radiological, and nuclear
ECTES: European Congress of Trauma and Emergency Surgery
LSI: Life-saving intervention
START: Simple Triage and Rapid Treatment
SALT: Sort, Assess, Life-saving interventions, Treatment/Transport
TASC: Tactical abbreviated surgical approach
MRMI: Medical Response to Major Incidents and Disasters Course
TDSC: Terror and Disaster Surgical Care Course
